# Dimeric assembly of F_1_-like ATPase for the gliding motility of *Mycoplasma*

**DOI:** 10.1126/sciadv.adr9319

**Published:** 2025-02-26

**Authors:** Takuma Toyonaga, Takayuki Kato, Akihiro Kawamoto, Tomoko Miyata, Keisuke Kawakami, Junso Fujita, Tasuku Hamaguchi, Keiichi Namba, Makoto Miyata

**Affiliations:** ^1^ Graduate School of Science, Osaka Metropolitan University, 3-3-138 Sugimoto, Sumiyoshi-ku, Osaka 558-8585, Japan.; ^2^ The OMU Advanced Research Institute for Natural Science and Technology, Osaka Metropolitan University, 3-3-138 Sugimoto, Sumiyoshi-ku, Osaka 558-8585, Japan.; ^3^ Institute for Protein Research, Osaka University, 3-2 Yamadaoka, Suita, Osaka 565-0871, Japan.; ^4^ Graduate School of Frontier Biosciences, Osaka University, 1-3 Yamadaoka, Suita, Osaka 565-0871, Japan.; ^5^ JEOL YOKOGUSHI Research Alliance Laboratories, Osaka University, 1-3 Yamadaoka, Suita, Osaka 565-0871, Japan.; ^6^ Biostructual Mechanism Laboratory, RIKEN, SPring-8 Center, 1-1-1, Kouto, Sayo, Hyogo 679-5148, Japan.; ^7^ Graduate School of Pharmaceutical Sciences, Osaka University, 1-6 Yamadaoka, Suita, Osaka 565-0871, Japan.; ^8^ Institute of Multidisciplinary Research for Advanced Materials, Tohoku University, 2-1-1 Katahira, Aoba-ku, Sendai 980-8577, Japan.

## Abstract

Rotary ATPases, including F_1_F_O_-, V_1_V_O_-, and A_1_A_O_-ATPases, are molecular motors that exhibit rotational movements for energy conversion. In the gliding bacterium, *Mycoplasma mobile*, a dimeric F_1_-like ATPase forms a chain structure within the cell, which is proposed to drive the gliding motility. However, the mechanisms of force generation and transmission remain unclear. We determined the electron cryomicroscopy (cryo-EM) structure of the dimeric F_1_-like ATPase complex. The structure revealed an assembly distinct from those of dimeric F_1_F_O_-ATPases. The F_1_-like ATPase unit associated by two subunits GliD and GliE was named G_1_-ATPase as an R_1_ domain of rotary ATPases. G_1_-β subunit, a homolog of the F_1_-ATPase catalytic subunit, exhibited a specific N-terminal region that incorporates the glycolytic enzyme, phosphoglycerate kinase into the complex. Structural features of the ATPase displayed strong similarities to F_1_-ATPase, suggesting a rotation based on the rotary catalytic mechanism. Overall, the cryo-EM structure provides insights into the mechanism through which G_1_-ATPase drives the *Mycoplasma* gliding motility.

## Teaser

A unique motor complex for motility of a parasitic bacterium was determined by cryo-EM, suggesting its mechanism and evolution.

## INTRODUCTION

**Fig. 1.**
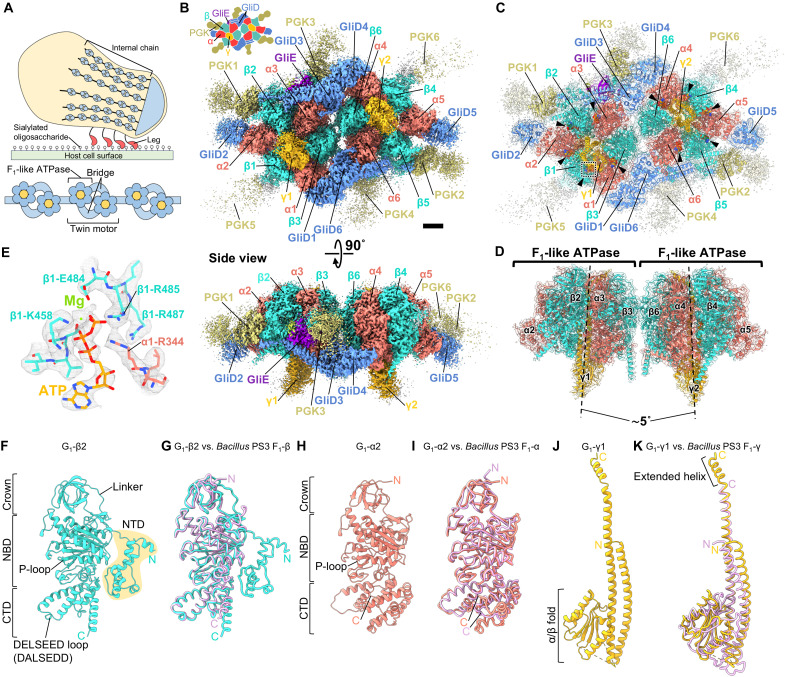
Structure of the twin motor featuring dimeric F_1_-like ATPase. (**A**) Gliding machinery of *Mycoplasma mobile*. Overall view of the gliding machinery (top) and part of the internal chain (bottom). (**B**) Overall structure of the twin motor. The illustration is drawn in the upper left. Scale bar, 25 Å. (**C**) Atomic model of the twin motor. Black triangles represent nucleotides. Maps in (B) and (C) are contoured at 0.55 in UCSF ChimeraX. (**D**) Dimeric F_1_-like ATPase in the twin motor. Dotted lines indicate the axis of each ATPase. (**E**) Density of Mg-ATP bound to G_1_-ATPase. The dotted region in (C) is shown. (**F**, **H**, and **J**) Subunits constituting F_1_-like ATPase (α_3_β_3_γ subcomplex of G_1_-ATPase). (**G**, **I**, and **K**) Overlay of subunits of G_1_-ATPase and *Bacillus* PS3 F_1_-ATPase. The subunits of the *Bacillus* PS3 F_1_-ATPase, αβ (PDB ID: 8HH5) and γ (PDB ID: 8HH2), are colored purple.

Rotary ATPases are molecular rotary motors that link adenosine 5′-triphosphate (ATP) hydrolysis or synthesis with ion transport via rotational motion in biological membranes (*1*). These motors comprise two common domains: soluble R_1_, which is responsible for ATP hydrolysis and synthesis, and membrane-embedded R_O_, which is responsible for ion transport. Rotary ATPase family defined by R_1_R_O_ includes F_1_F_O_-, V_1_V_O_-, and A_1_A_O_-ATPases (*1*). Notably, F_1_F_O_-ATPase, also known as F_1_F_O_ ATP synthase, is conserved across most eukaryotes, archaea, and bacteria and responsible for ATP synthesis and the maintenance of membrane potential (*2*). An F_1_-like ATPase gene cluster, referred to as type 2 ATPase, was reported in four *Mycoplasma* species, *Mycoplasma mobile*, *M. pulmonis*, *M. agassizii*, and *M. testudineum*, in addition to the genuine F_1_F_O_-ATPase gene cluster, referred to as type 1 ATPase (*3*, *4*) (fig. S1). The presence of F_1_-ATPase α and β subunit homologs (MMOBs 1660 and 1670 in *M. mobile*) in the type 2 ATPase gene cluster suggests a shared evolutionary origin in the F_1_ domain. Type 2 ATPase is proposed to drive the gliding motility observed in *M. mobile* and *M. pulmonis* (*5*–*9*). *M. mobile*, a fish pathogen, glides at speeds of up to 4 μm per second on a solid surface covered with sialylated oligosaccharides found on host cell surfaces (*10*) (Fig. 1A and fig. S2). In our proposed model for gliding, filamentous leg structures on the mycoplasma cell surface, which are approximately 50 nm, propel the cell body by catching and pulling sialylated oligosaccharides on the solid surface. Inside the cell, motors composed of type 2 ATPase and phosphoglycerate kinase (PGK), which converts adenosine 5′-diphosphate (ADP) to ATP through glycolysis, are linked near the cell membrane to form “internal chains” (*7*, *11*). The motor is formed by dimerized F_1_-like ATPases with unique bridge structures and is referred to as a twin motor (*7*, *12*). However, the mechanisms of force generation and transmission remain unclear. This study aimed to elucidate the detailed structure of the twin motor using electron cryomicroscopy (cryo-EM) single-particle analysis (SPA). Notably, the structural features had a strong homology to those of F_1_-ATPase, suggesting a rotary catalytic mechanism analogous to that of F_1_-ATPase (*1*, *13*–*16*) for the gliding motility of *M. mobile*.

## RESULTS

### Protein identification using the cryo-EM structure of twin motor

Cryo-EM SPA was performed for twin motors isolated from *M. mobile* cells using images collected on the epoxidized graphene grid (EG-grid) (*17*) (table S1). The three-dimensional (3D) structure of the twin motor was determined at a resolution of 3.2 Å (Fig. 1B and figs. S3 and S4). Local refinement improved the resolution of the regions, each containing the F_1_-like ATPase unit, to 3.1 Å (figs. S3B and S4). The density maps enabled the construction of the atomic model for most regions of the twin motor (Fig. 1C). The modeled sequence regions of each subunit are summarized in table S2. The twin motor exhibited a rectangular structure with dimensions of approximately 350 × 250 × 150 Å and at least 24 polypeptide chains with a total mass of approximately 1.5 MDa. Notably, the two F_1_-like ATPases were inclined to each other by approximately 5° and formed a dimer with pseudo-twofold rotational symmetry and several accessories (Fig. 1, C and D). The F_1_-like ATPase comprised a hexameric ring of two alternating subunits and a central shaft subunit, identified as MMOBs 1660, 1670, and 1630, respectively. The conserved ATPase structure of the R_1_ domain in a unique motor indicates that the F_1_-like ATPase clarified here is a new R_1_ domain. In the present study, the ATPase unit was designated G_1_-ATPase, where “G” is derived from “gliding,” with assignment of MMOBs 1660, 1670, and 1630 to α, β, and γ, respectively, according to the subunit names of F_1_-ATPase (*14*). In the hexameric ring, nucleotide-derived densities were observed at five of the six subunit interfaces, despite the isolation and analysis of samples under nucleotide free condition, indicating the presence of endogenous nucleotides as reported in F_1_-ATPase (*18*) (Fig. 1, C and E). The subunit attached to α, forming a “bridge” between two α_3_β_3_γ subcomplexes, was identified as MMOB1620 and named GliD in the present study (Fig. 1C). The three densities protruding from the G_1_-ATPase dimer were identified as MMOB4530, which was previously annotated as PGK. Because of the low resolution of the PGK1–3 regions at the edge of the map, atomic models were constructed for only 204, 203, and 203 amino acids of the total length of 511 amino acids, including 60–, 72–, and 98–amino acid alanine truncations, respectively (table S2 and fig. S5). The denoised 3D map revealed additional distinct densities at six locations, including the PGK1–3 regions (fig. S6A). These six densities exhibited correlation coefficients ranging from 0.37 to 0.48 with the crystal structure of PGK from *Staphylococcus aureus* [Protein Data Bank (PDB) ID: 4DG5] (fig. S6, B and C). Although the values are low, these assignments are consistent with the stoichiometry previously estimated via SDS–polyacrylamide gel electrophoresis (PAGE) (*7*), suggesting that these maps are PGK molecules. A density bound only to one of the two α_3_β_3_γ subcomplexes was identified as MMOB3660, which was newly detected via SDS-PAGE of the twin motor and named GliE as a G_1_-ATPase subunit in the present study (Fig. 1, B and C, and fig. S7). This small protein (112 amino acids), characterized by a β-sandwich, was nestled between the ATPase hexamer and other subunits, breaking the rotational symmetry of the complex (fig. S8). Although MMOB1640, encoded in the type 2 ATPase gene cluster, was detected via SDS-PAGE of the twin motor (fig. S7), the protein density was not observed from the overall 3D map.

### Structural features of G_1_-ATPase αβγ

**Fig. 2.**
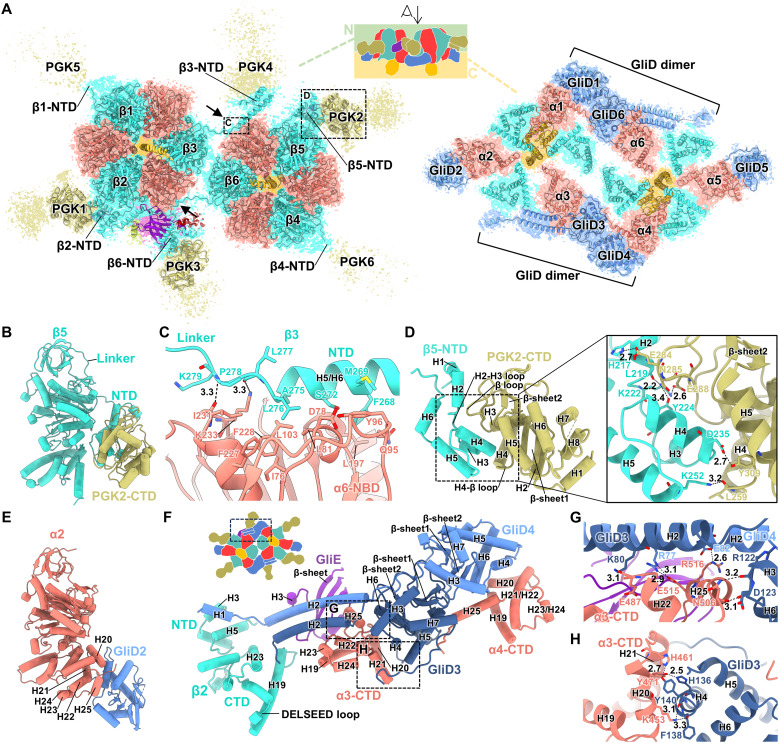
Interaction between subunits. (**A**) N- and C-terminal regions of the twin motor. The twin motor is viewed from the top of the illustration. Arrows indicate the interface between G_1_-ATPase monomers. Maps are contoured at 0.55 in UCSF ChimeraX. (**B**) Structure of β and PGK. (**C**) Interaction between the N-terminal region of β and α-NBD at the interface between G_1_-ATPase monomers. The dotted region in (A) is shown. (**D**) Interaction of PGK with the β-NTD. The dotted region in (A) is shown. (**E**) Structure of the α and GliD monomers. (**F**) GliD dimer at the interface between G_1_-ATPase monomers. The figure corresponds to the dotted region in the illustration. (**G** and **H**) Interaction between GliD and α-CTD. Each corresponds to the dotted regions in (F).

G_1_-β and G_1_-α exhibited high structural similarity to the β and α subunits of the F_1_-ATPase from the thermophilic bacterium, *Bacillus* PS3 (PDB ID: 8HH5), based on the superimposition of their Cα atoms with the root mean squared deviation (RMSD) values of 1.95 and 2.79 Å, respectively (Fig. 1, G and I). Both G_1_-β and G_1_-α had three domains: crown, nucleotide-binding domain (NBD), and C-terminal domain (CTD) (Fig. 1, F and H). The NBDs of G_1_-β and G_1_-α comprise a phosphate-binding motif of P-loop (*19*), consisting of the amino acid sequences GGAGVGKT and GDRGTGKT, respectively. The G_1_-β-CTD contained a DELSEED loop, which is important for torque transmission in the F_1_-ATPase β subunit (*20*), with an amino acid sequence of DALSEDD (Fig. 1F). A remarkable difference between G_1_-β and the F_1_-ATPase β subunit was the presence of an extended N-terminal region consisting of the N-terminal domain (NTD) and linker (Fig. 1F and fig. S9A). This region comprised approximately 100 residues located within the extra N-terminal 299 residues of G_1_-β (*4*, *11*). Notably, the linker hangs outside the G_1_-ATPase along the crown and NBD of G_1_-β (fig. S9B). G_1_-β-NTDs, consisting of four to six helices, were oriented in various directions among G_1_-β, depending on their position in the complex (fig. S9, C and D). Further, G_1_-β-NTD interacted with PGK (Fig. 2, A and B). In addition, at the interface of the two G_1_-ATPase monomers, the N-terminal regions of G_1_-β3 and G_1_-β6 interacted with the NBDs of G_1_-α6 and G_1_-α3 of another G_1_-ATPase monomer, respectively (Fig. 2, A and C). These interactions suggest that the β-specific N-terminal region plays a role in the incorporation of PGK into the twin motor and stabilization of the ATPase dimer. A difference between G_1_-α and the F_1_-ATPase α subunit is the absence of the H1 helix, which interacts with the peripheral stalk (*21*) (fig. S10). This absence is consistent with the lack of a gene homologous to the peripheral stalk in the type 2 ATPase gene cluster (*4*). G_1_-γ, which is composed of a coiled coil and an α/β fold typical of the F_1_-ATPase γ subunit, was superimposed on the γ subunit of the *Bacillus* PS3 F_1_-ATPase with an RMSD value of 5.79 Å for Cα atoms due to a lower sequence conservation than that of the catalytic subunits (*10*) (Fig. 1, J and K). The folded C-terminal α-helix, which is 25 Å longer than the F_1_-ATPase γ subunit, protrudes from the N-terminal side of the hexameric ring and interacts with the crowns of G_1_-αβ (Fig. 1K and fig. S11, A and B). The coiled coil of G_1_-γ hydrophobically interacted with the CTDs of G_1_-αβ in the hexameric ring (fig. S11C), and in the F_1_-ATPase, this interaction is involved in axle hold and torque transmission (*22*).

### Accessories of ATPase dimer

In general, PGK is bifurcated into two domains interconnected by an α-helix: the NTD, which binds to 3-phosphoglycerate (3-PG) or 1,3-bisphosphoglycerate (1,3-BPG), and the CTD, which binds to Mg-ADP or Mg-ATP (*23*) (fig. S12A). The twin-motor PGK structure, comprising the CTD and a segment of the α-helix, exhibited a high structural similarity to the PGK from *S. aureus* (PDB ID: 4DG5), with an RMSD value of 3.12 Å for Cα atoms (fig. S12, B and C). The β loop of the PGK-CTD invades the depression of the folded β-NTD and interacts with the H2 helix and the H2–H3 loop of the β-NTD (Fig. 2D). The H4–β loop and H5 helix of the PGK-CTD also interacted with the H5 and H4 helices of the β-NTD, respectively. Nucleotide-derived densities were not found in the PGK-CTD of the twin motor, despite conservation of the residues important for nucleotide binding in PGK (*24*) (figs. S12, D to F, and S13). None of the twin-motor PGK-NTDs were modeled due to the poor densities of the reconstruction. However, the binding and catalytic residues for 3-PG or 1,3-BPG in PGK were conserved in terms of amino acid sequence, suggesting that the twin-motor PGK has enzymatic activity (fig. S13). As the binding and catalytic residues were not conserved in another PGK homolog, MMOB4490, encoded in the *M. mobile* genome, the twin-motor PGK is expected to function as the original PGK in glycolysis (fig. S13). The GliD (GliD1,3,4,6) bridge components formed dimers at the interface of two G_1_-ATPase monomers, whereas GliD2,5 at the twin-motor end existed as monomers (Fig. 2, A, E, and F). GliD contained a globular domain and an N-terminal long α-helix (H2), which was only visualized in the dimer (Fig. 2F). The globular domain interacted with the H20–H25 region at the C terminus of α (Fig. 2, G and H). The two H2 helices in the GliD dimer aligned parallel to each other across the H20–H25 region of α, GliE, β-NTD, and β-CTD (Fig. 2F). These two α-helices are reminiscent of the dynein buttress that connects the AAA+ ring and the tubulin-binding domain (*25*), suggesting a regulatory role for structural changes induced by the ATPase activity of the G_1_-ATPases.

### Structural information suggesting a catalytic mechanism analogous to that of F_1_-ATPase

**Fig. 3.**
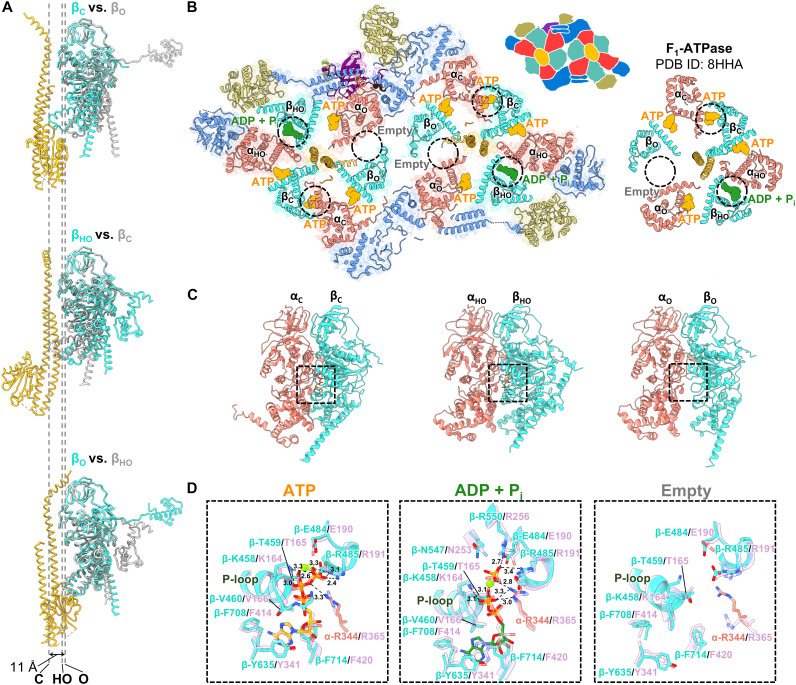
Structural conformations and nucleotide binding of β. (**A**) Differences in the conformations of the three β. Superposed three βs in the G_1_-ATPase. The dotted lines indicate the position of the DELSEED loop of each β. (**B**) Nucleotide binding pattern of dimeric G_1_-ATPase and F_1_-ATPase. The catalytic site is circled by black dotted lines. The illustration of the twin motor is drawn in the upper right. (**C**) αβ dimer in each conformation in the hexamer. (**D**) Comparison of the catalytic sites. Each catalytic site corresponds to the dot square above. The catalytic sites are superimposed on the corresponding catalytic sites of F_1_-ATPase (PDB ID: 8HHA), which are colored purple.

The crown region of the hexameric ring exhibited approximately sixfold rotational symmetry, whereas the CTD region was asymmetric (fig. S11A). This observation suggests a structural change with a pivot point between the crown and CTD. β1–3 adopted three distinct conformations, which appear to be the transition states of CTD flexion motion toward γ, with a positional difference of 11 Å for the DELSEED loop (Fig. 3A). These conformations were named β_C_ (closed), β_HO_ (half-open), and β_O_ (open), according to the conformation names of F_1_-ATPase (*16*). The conformations were arranged clockwise when viewed from the C terminus of the hexameric ring (Fig. 3B). These conformations resemble the states referred to as ATP-waiting or step-waiting, which occurs during the catalytic reaction of *Bacillus* PS3 F_1_-ATPase; the latter occurs after ATP binding (*16*) (table S3). In α, the region corresponding to the DELSEED loop exhibited only a 5 Å difference in position relative to the γ side, whereas the H20–H25 region interacting with GliD exhibited a difference of 9 Å (fig. S14). These conformations were named α_C_ (close), α_HO_ (half-open), and α_O_ (open) based on the H20–H25 positions viewed from the γ side. If this difference in α represents a conformational change associated with ATP hydrolysis of the twin motor, force transfer may occur from the α_3_β_3_γ subcomplex of G_1_-ATPase to GliD. The catalytic sites formed at the α-β interface of α_C_β_C_ and α_HO_β_HO_ contained Mg-ATP and Mg-ADP + P_i_, respectively, whereas no nucleotide density was found at the catalytic site of α_O_β_O_ (Fig. 3, B to D, and fig. S15). The nucleotide-binding states were also shared between the two G_1_-ATPase monomers according to the pseudo-twofold rotational symmetry. The residues of F_1_-ATPase involved in ATP hydrolysis, including those in the arginine finger, were conserved in G_1_-αβ, suggesting a shared ATP hydrolysis pathway between F_1_-ATPase and G_1_-ATPase (Fig. 3D). Mg-ATP was observed at the remaining three interfaces, hereafter referred to as the β-α interfaces (Fig. 3B and fig. S15). At the β-α interface, α-K179 of G_1_-ATPase corresponded to the position of conserved residue Q200 of the F_1_-ATPase α subunit (fig. S16, A and B). Compared with the α-β interface, α-K179 is charge inverted relative to the glutamate essential for the catalytic activity (E190 of the F_1_-ATPase β subunit), suggesting that the β-α interface, akin to F_1_-ATPase, does not exhibit ATP hydrolysis activity (fig. S16C). The conformational sequence pattern of three β subunits with bound nucleotides was identical to that of the ATP-waiting state of F_1_-ATPase (*16*) (Fig. 3B). These data suggest that Mg-ATP binding to the catalytic site of α_O_β_O_ promotes the catalytic reaction of the G_1_-ATPase based on the catalytic mechanism analogous to that of F_1_-ATPase, as discussed below.

### Twin motors in the chain

**Fig. 4.**
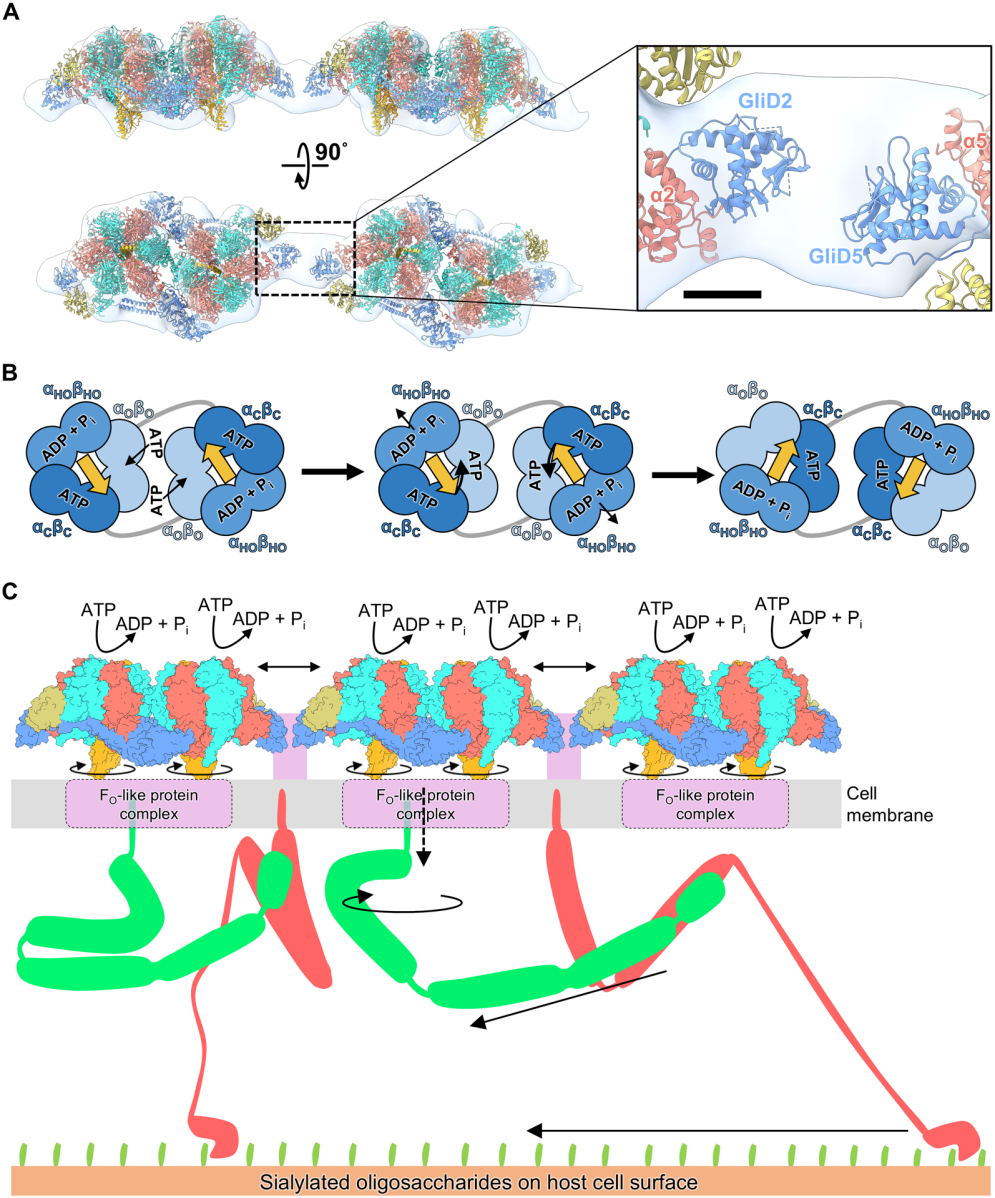
Structure and motion of the gliding machinery. (**A**) Fitting of the atomic model of the twin-motor into the EM map of the internal chain. The right panel indicates the interface between GliDs. Scale bar, 25 Å. (**B**) Possible rotational catalytic mechanism of G_1_-ATPase. The 120° rotation of γ coupled with the conformational change in the catalytic hexamer is explained, based on the tri-site mechanism of F_1_-ATPase (*16*). γ is indicated by yellow arrows, the direction of which indicates the conformational tilt. (**C**) A possible explanation for the conversion mechanism from rotational to linear motion. The regions with asymmetric structures are excluded from the internal chain model as done in (A). Dotted arrows indicate unknown force transduction in the cell membrane. Double-headed arrows indicate the changes in the distance between twin motors. Gli349 and Gli521 are depicted in red and green, respectively. Unidentified protein regions in the gliding machinery are colored purple.

The atomic model of the twin motor comprising G_1_-ATPase dimer and PGK was fitted into the previously reconstructed density of the internal chain, obtained using negative staining electron microscopy (EM) SPA (*7*) (Fig. 4A). To circumvent misfitting of the long-axis orientation of the twin motors due to the low-resolution density map (29.7 Å) of the chain structure, regions with asymmetric structures, such as the NTDs of β3,6, GliE, and PGK3, were excluded from the fitted model. At the interface between twin-motor units, GliDs that project along the long axis of the twin motor faced each other, suggesting their role as a linker for chain formation. However, the 3D map of the internal chain revealed a remaining density at the interface that cannot be filled by GliDs, which may correspond to the approximately 100 residues comprising an N-terminal region and loops that have not been modeled.

## DISCUSSION

The major difference between the G_1_-ATPase dimer and the dimeric form of eukaryotic mitochondrial F_1_F_O_-ATPases (*2*) is the interaction between the R_1_ domains through GliD and the N-terminal region of β. Although the GliD dimer does not have a transmembrane segment, the two long α-helices at the N terminus are reminiscent of the structure of a peripheral stalk. This observation is consistent with the hypothesis that the MMOB1620 gene encoding GliD is derived through gene fusion from the b and δ subunits, which comprise the peripheral stalk (*10*). The GliD-mediated dimer and chain formation may allow G_1_-ATPases to work cooperatively. Consistently, changes in the distance between twin motors have been associated with the ATP hydrolysis reaction, suggesting force transfer between the twin motors through GliD (*5*, *6*). The connections between α_3_β_3_ catalytic hexamers in the G_1_-ATPase dimer may be related to the lower ATPase activity than that of the F_1_-ATPase, with a maximum turnover rate of 0.18 s^−1^ per β molecule and a *K*_m_ of 74 μM (*7*). The twin-motor PGK may regulate the assembly and activity of ATPases, as observed for 6-phosphofructo-1-kinase and aldolase, glycolytic enzymes that bind to V_1_V_O_-ATPase (*26*, *27*). Further, PGK molecules protruding from the transverse axis of the twin motor may be involved in the sheet formation of the internal chain, according to negative staining EM (*12*). The weak density of PGK in the cryo-EM map can be explained by its flexibility, which was identified using high-speed atomic force microscopy (*7*). The flexibility of the PGK molecule may suggest that the chain sheet serves as a cytoskeleton to withstand mechanical stress in the mycoplasma cell, which does not have a cell wall. The GliE subunit, which is bound to only one side of the dimeric G_1_-ATPase, creates asymmetry in the twin motor structure and may contribute to the unidirectional nature of the gliding motility. However, this protein may also bind to another side of the dimeric G_1_-ATPase in the cell but dislodge during purification.

The three distinct forms of the catalytic subunit β and their corresponding nucleotide binding suggest a rotary catalytic mechanism analogous to that of F_1_-ATPase (*16*) (Fig. 4B). In this rotary catalytic mechanism, α_O_β_O_ transitions to α_C_β_C_ after ATP binding at the catalytic site, and the DELSEED loop of β in this transition approaches γ. The other αβ dimers in the hexameric ring also operate cooperatively, with α_C_β_C_ transitioning to α_HO_β_HO_, associated with ATP hydrolysis and α_HO_β_HO_ transitioning to α_O_β_O_, associated with the release of ADP and phosphate. This one-step conformational change of the hexameric ring prompts a 120° rotation of γ. Each αβ dimer sequentially hydrolyses ATP, and through a three-step process, the hexameric ring structure and position of γ revert to their initial states. The pseudo-twofold rotational symmetry structure of the twin motor suggests that the two G_1_-ATPase monomers operate in a synchronized manner, maintaining the structural symmetry. Throughout the catalytic reaction, substantial changes in the positioning of the two G_1_-ATPase monomers within the twin motor may be hindered due to the presence of the N-terminal regions of β bridging the two monomers and a buttress-like structure composed of two long α-helices of the GliD dimer (Fig. 2, C and F).

For cell gliding, the rotary motions of the twin motors must be converted into a linear motion. This conversion mechanism may occur due to the membrane protein Gli521, which consists of a hook and rod, thereby functioning as a crank (*28*) (Fig. 4C). Upon the conversion of the rotary motion to linear motion, Gli521 pulls the leg protein, Gli349, which is bound to sialylated oligosaccharides on the host cell surface, thereby propelling the mycoplasma cell forward (*29*). The presence of a globular domain in G_1_-γ, similar to that in the F_1_-ATPase γ subunit, suggests a potential interaction with a membrane-embedded F_O_-like domain. MMOB1610 and MMOB1650, encoded in the type 2 ATPase gene cluster, have 12 and 2 transmembrane segments, respectively (*10*) (fig. S1). These two proteins are potential interaction partners for the globular domain of γ at the cell membrane, transmitting the rotary motion to Gli521 on the cell surface.

The roles of rotary ATPases and their relatives, such as the flagellar type III protein export apparatus, which exhibit a hexameric ring with a central shaft, involve protein and ion transport (*30*, *31*). We hypothesized that F_1_F_O_-ATPase has acquired the role and mechanism of the twin motor through an incidental contact with an adhesin on the membrane during the evolution of mycoplasma from a nonmotile to a motile state (*7*, *32*). This primitive gliding motility would have conferred a survival advantage. Another F_1_-like ATPase, referred to as type 3 ATPase, is also found in mycoplasma, and this F_1_-like ATPase may drive the system that cleaves host antibodies (*4*, *33*). Notably, types 2 and 3 ATPases transition from F_1_-ATPase to drive mycoplasma-specific systems. Understanding these unique ATPases will contribute to investigations into the working principles and evolution of rotary ATPases.

## METHODS

### Optical microscopy

The cells of the *M. mobile* mutant strain [gli521 (P476R)], which can glide like the wild-type strain but binds sialylated oligosaccharides more tightly, were cultured, observed using phase-contrast microscopy, and recorded as previously described (*34*–*37*). The recorded video was analyzed using ImageJ software version 1.54d (https://imagej.nih.gov/ij/).

### Isolation of the twin motor

The twin motor was isolated from the *M. mobile* mutant strain [gli521 (P476R)], as previously described (*7*). The twin motor sample with phosphate-buffered saline consisting of 8.1 mM Na_2_HPO_4_, 1.5 mM KH_2_PO_4_ (pH 7.3), 2.7 mM KCl and 137 mM NaCl, and 1 mM MgCl_2_ was concentrated to 1 mg/ml using a 100-K Vivaspin concentrator (Merck, Germany) with 0.1% (w/v) CHAPS to prevent membrane adsorption. The concentrated twin motor was subjected to 12.5% SDS-PAGE and stained using Coomassie brilliant blue R-250. The bands of the component proteins were identified using peptide mass fingerprinting, as previously described (*38*).

### Cryo-EM grid preparation and data acquisition

An EG-grid was prepared as previously described (*39*). A 2.6-μl sample solution was applied on the grid, automatically blotted from both sides with filter paper at 4°C for 2 s at 100% humidity, and vitrificated using the semi-automated vitrification device, Vitrobot Mark IV (Thermo Fisher Scientific, USA). Cryo-EM imaging was performed using a CRYO ARM 300 (JEOL, Japan) operated at 300 kV with a K3 direct electron detector camera (AMETEK, Gatan, USA). A total of 7350 movies were recorded using SerialEM software (*40*) with a total dose of approximately 80 electrons Å^−2^ for 40 frames, an exposure time of 3.3 s per movie, and a nominal defocus range of −0.8 to −1.8 μm. The nominal magnification was 60,000×, corresponding to 0.87 Å per pixel.

### Cryo-EM image processing

The image processing steps for SPA are summarized in figs. S3 and S4. Image processing was performed using cryoSPARC version 3.3.2 (*41*), unless otherwise stated. Movies were aligned using patch-based motion correction, and the contrast transfer function (CTF) parameters were estimated using patch CTF estimation. Micrographs with a CTF fit resolution worse than 8 Å were removed. Particles were automatically picked up using a Blob Picker, extracted with ×4 binning, and 2D classified. The obtained 2D averaged images were used as templates for the Template Picker to pick up good particles. After two rounds of 2D classification, the selected good particles were subjected to local motion correction and re-extracted with the original pixel size. The particles were subjected to ab initio reconstruction using C1 symmetry enforced with a final resolution limit of 12 Å and heterogeneous reconstruction with three classes. Particles in class 2 were subjected to homogeneous reconstruction using C1 symmetry, resulting in a map at a resolution of 3.5 Å. The final map was generated via local and global CTF refinement, and then nonuniform refinement. To improve the local resolution, the final map and particles were subjected to particle subtraction and local refinement using C1 symmetry to generate half-divided maps, each containing a monomeric ATPase. The whole and two local maps were subjected to mask creation and postprocessing in RELION 4.0 (*42*), resulting in resolutions of 3.2, 3.1, and 3.1 Å, respectively. Local resolution estimation was also performed in RELION 4.0. The postprocessed map was denoised using Topaz software version 0.2.5 with the trained model Unet-3d-10a (*43*). RELION 4.0 was used to calculate the Fourier shell correlation (FSC) curve between two half-maps and estimate the local resolution.

### Modeling

Initial models for α, β, γ, GliD, and GliE were generated using AlphaFold2 (*44*), whereas the homology model for PGK was generated using Modeller version 10.4 (*45*) with the atomic model of PGK (PDB ID: 4DG5) from *S. aureus*. The generated models were fitted to the corresponding densities of the EM map as rigid bodies using UCSF Chimera version 1.15 (*46*). The fitted models were manually inspected and adjusted using COOT version 0.9.8.1 (*47*) and refined using the phenix.real_space_refine program in PHENIX version 1.19 (*48*, *49*) with reference model restraints using atomic models of F_1_-ATPase (PDB ID: 5IK2) from *Caldalaklibacillus thermarum* and PGK (PDB ID: 3ZLB) from *Streptococcus pneumoniae*. Manual adjustment using COOT and refinement using PHENIX were repeated until model parameters were no longer improved. For the modeling of PGK3, the atomic model of PGK1 was fitted to the PGK3 map and then manually inspected using COOT. FSC curves between the map and model were calculated using PHENIX. The refined model was also evaluated via comprehensive validation in PHENIX and the Q-score (*50*). To identify the nucleotide binding sites of G_1_-ATPases, F_o_-F_c_ maps were generated using Servalcat (*51*). Superposition of models and fitting of models to the denoised or negative staining EM map were performed using UCSF ChimeraX version 1.2.5 (*52*). All structural figures were obtained using UCSF ChimeraX.
