# Supplementary material for: Dimeric assembly of F1-like ATPase for the gliding motility of Mycoplasma
Source: Sci Adv. 2025 Feb 26;11(9):eadr9319. doi: 10.1126/sciadv.adr9319 (PMC11864180; doi:10.1126/sciadv.adr9319)
Supplement: Supplementary file 1 — Figs. S1 to S16 Tables S1 to S3 [file sciadv.adr9319_sm.pdf]

Science Advances
AAAS

Supplementary Materials for

Dimeric assembly of F_1-like ATPase for the gliding motility of *Mycoplasma*

Takuma Toyonaga *et al.*

Corresponding author: Makoto Miyata, miyata@omu.ac.jp

Sci. Adv. **11**, eadr9319 (2025)
DOI: 10.1126/sciadv.adr9319

This PDF file includes:

Figs. S1 to S16
Tables S1 to S3

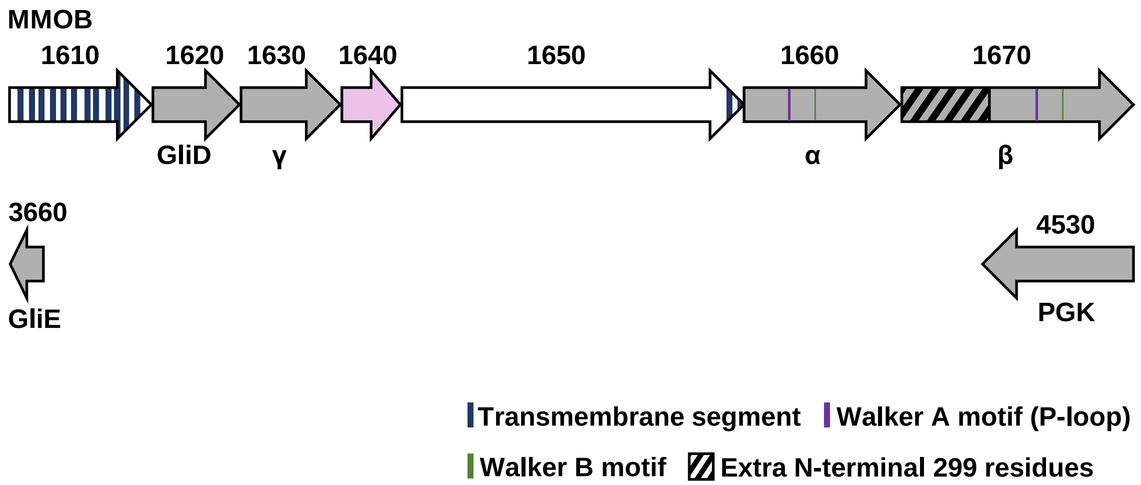

Fig. S1. Open reading frames related to twin motor in *M. mobile*.
The Type 2 ATPase cluster is composed of MMOBs 1610–1670. The twin-motor components are shown in gray. In this study, MMOB1640, coloured in pink, was suggested as a component only by SDS-PAGE analysis.

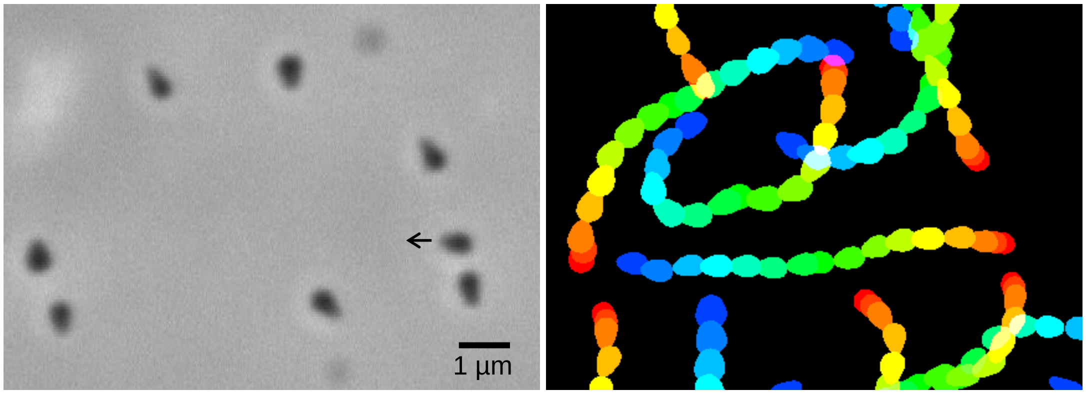

Fig. S2. Optical microscopy of *M. mobile* gliding.
The left panel shows the cell image. Arrow indicates the gliding direction. The right panel shows the rainbow trace of *M. mobile* gliding. The gliding cells were traced every 0.2 s and stacked for 3 s. The trajectories are coloured from red to blue.

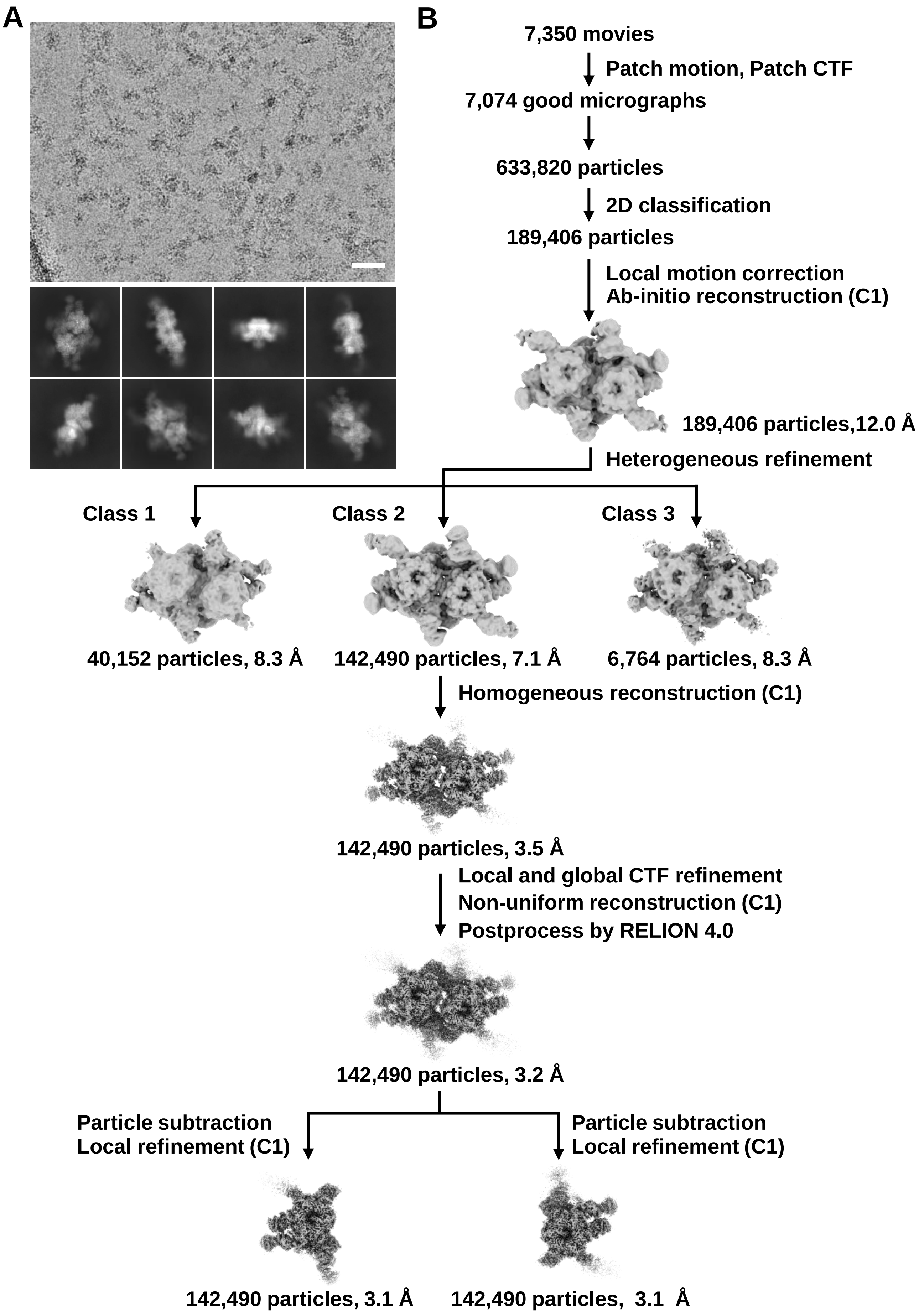

Fig. S3. Image processing.
(**A**) Representative micrograph (top) and averaged images (bottom). Scale bar, 500 Å. (**B**) Workflow of image processing.

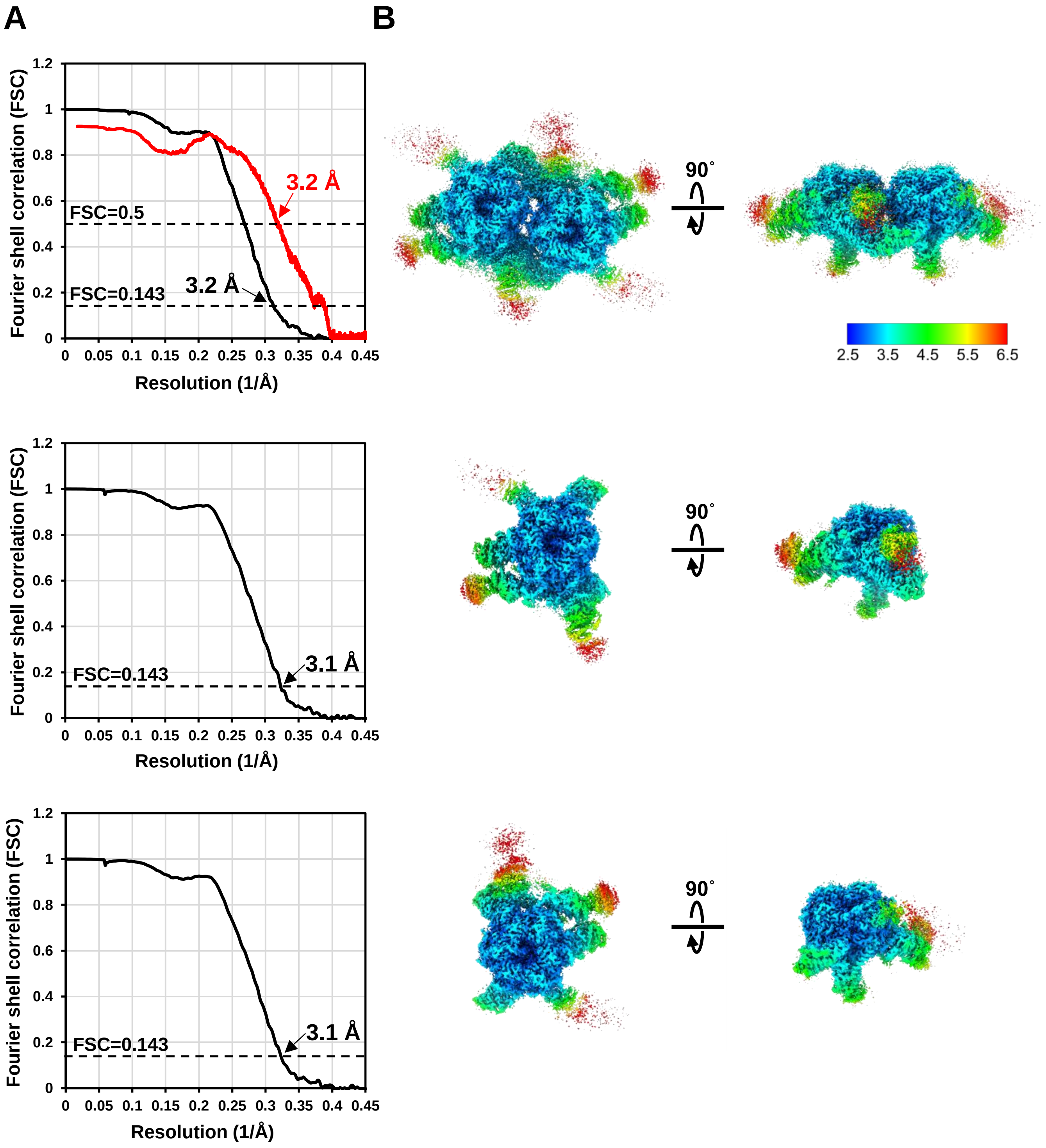

Fig. S4. Fourier shell correlation and local resolution estimation of density maps.
(**A**) Fourier shell correlation curves for half maps (black) and cross-validation between the density map and model (red). (**B**) Local resolution estimated using RELION.

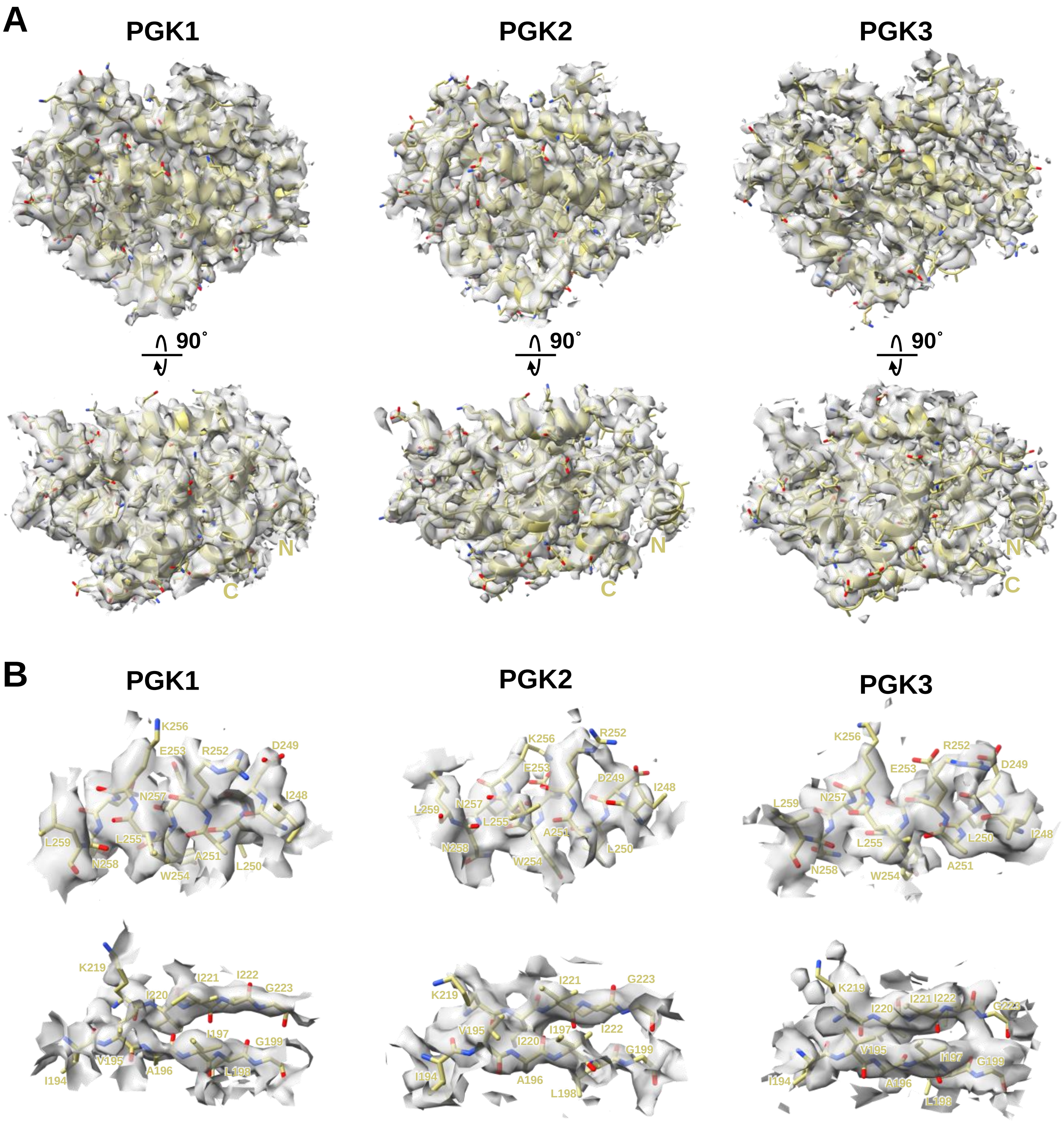

Fig. S5. Density maps and models of PGK molecules.
(**A**) Maps after local refinement and atomic models of PGK molecules. (**B**) Modelled α-helices and β-sheets. The maps in (A) and (B) are contoured at 0.42 except for PGK3 (0.32) in UCSF ChimeraX.

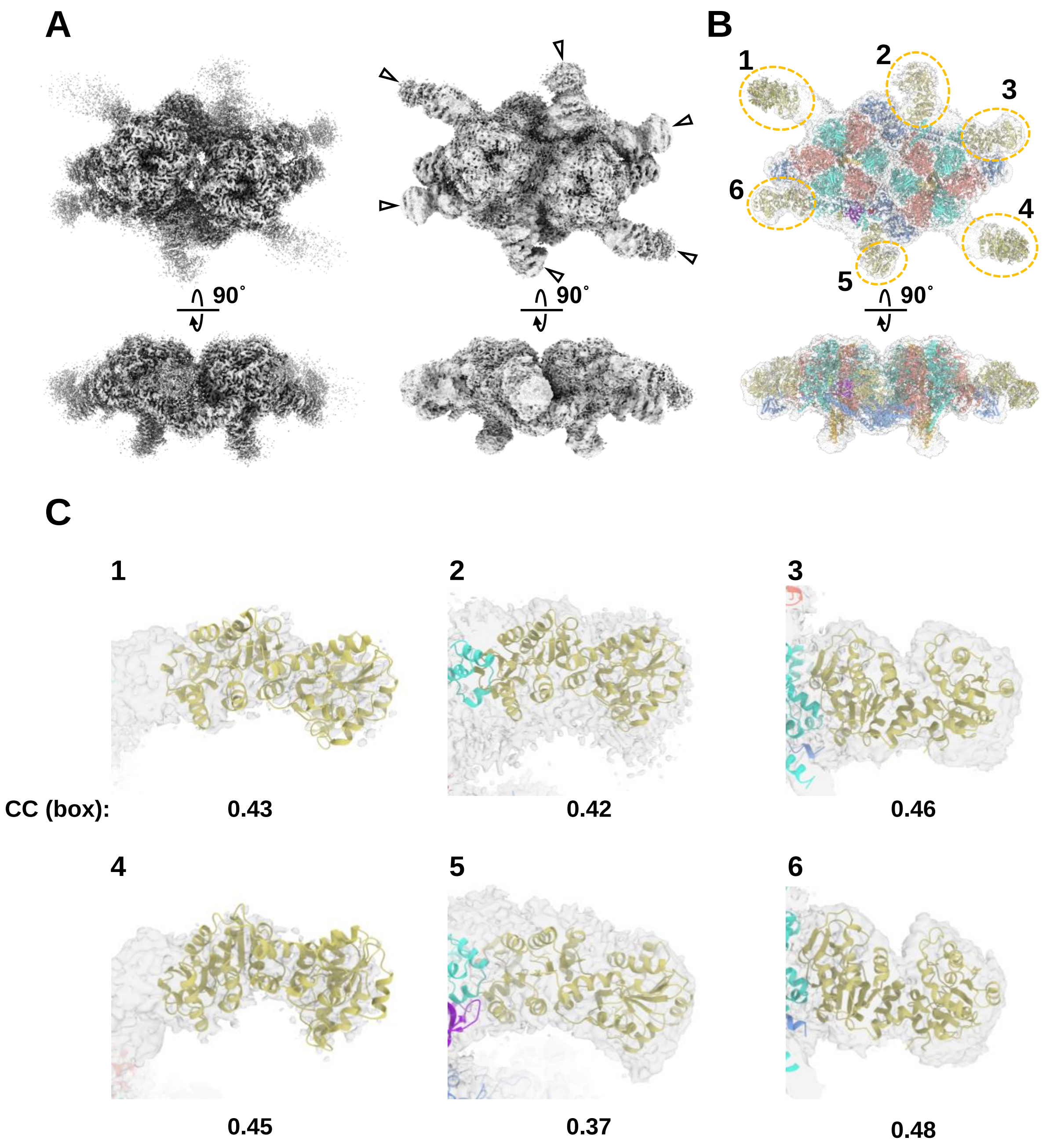

Fig. S6. Densities corresponding to PGK molecules.
(**A**) Comparison between maps before and after denoising (left and right). Densities due to denoising are indicated by triangles. The maps before and after denoising are contoured at 0.55 and 0.12 in UCSF ChimeraX. (**B**) Denoised map fitted with atomic models of twin-motor subunits and PGKs from *S. aureus* (PDB ID: 4DG5). Densities corresponding to the PGK molecules were marked with orange dot circles. (**C**) Fitting of PGK molecules; numbers correspond to (B).

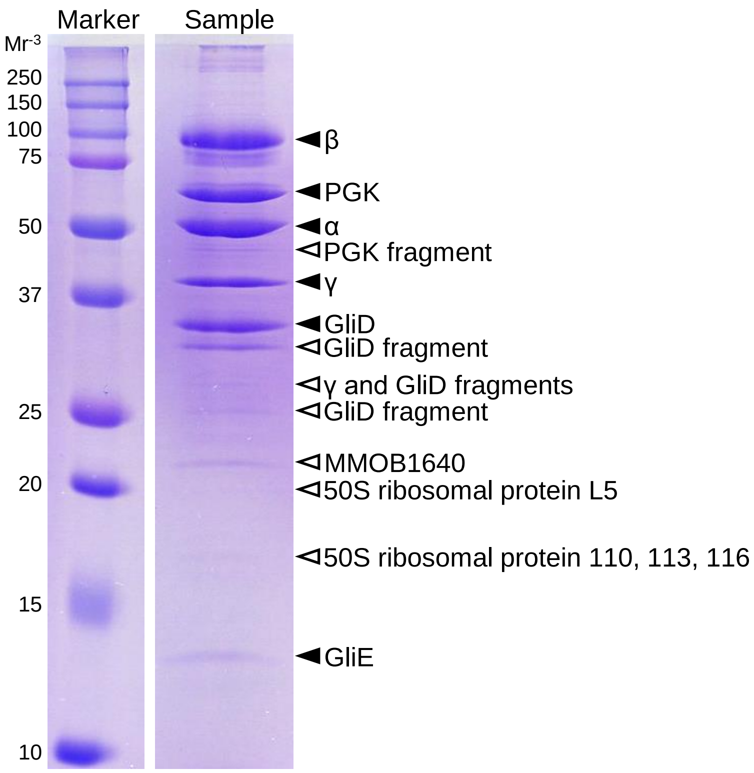

Fig. S7. SDS-PAGE of isolated twin motor.
The sample was subjected to 12.5% SDS-PAGE gel and stained with Coomassie brilliant blue R-250. Twin-motor components are indicated by black triangles. Minor protein bands in the sample fraction were identified as marked with white triangles. Molecular masses are shown on the left.

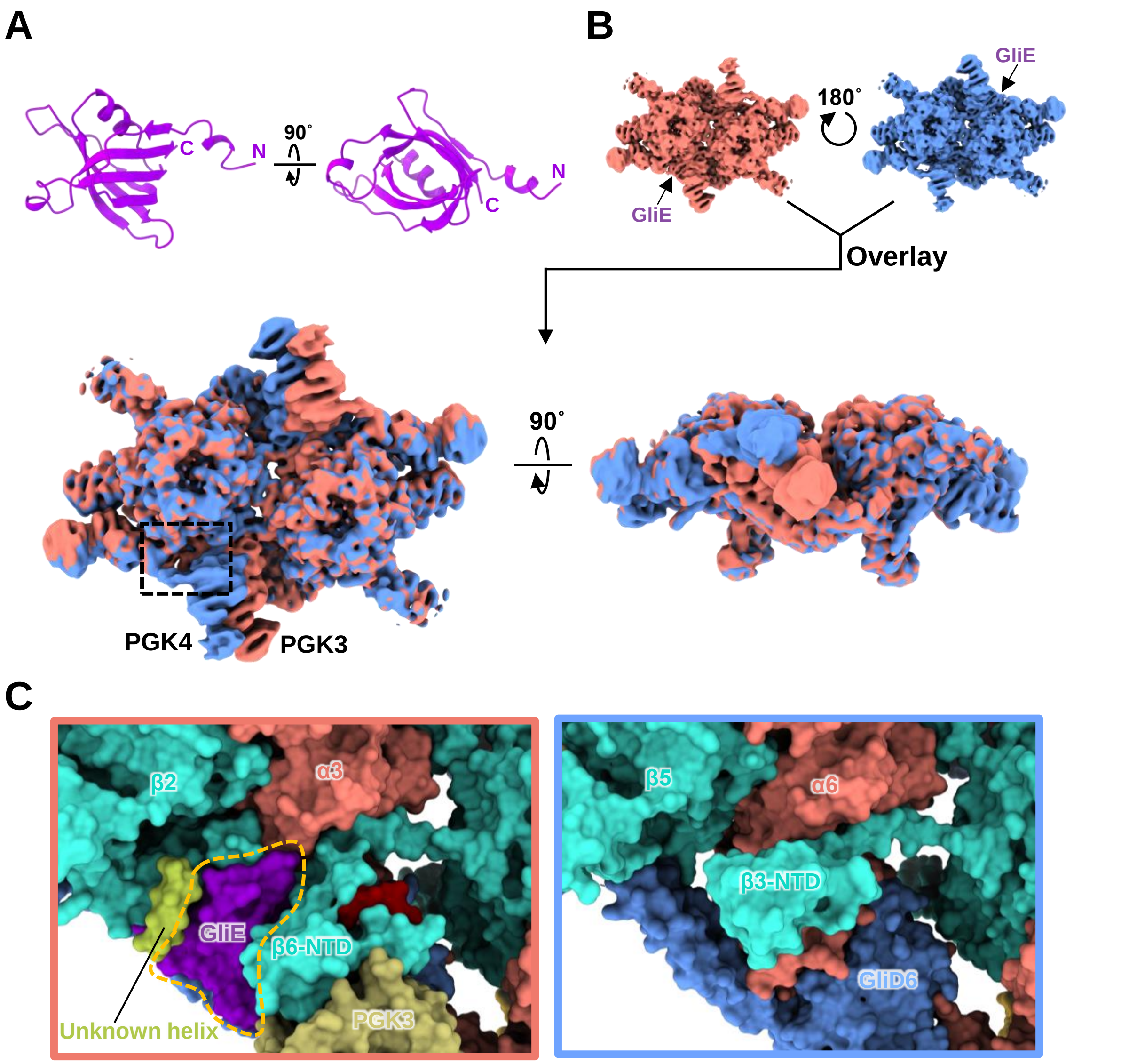

Fig. S8. Asymmetry of twin-motor structure.
(**A**) GliE structure. (**B**) Overlay of the twin-motor structures rotated by 180˚. The maps were low-pass filtered at 8 Å in RELION and contoured at 0.17 in UCSF ChimeraX. (**C**) Comparison of rotated twin-motor structures. The dotted square area in (B) is shown. GliE is surrounded by an orange dot line.

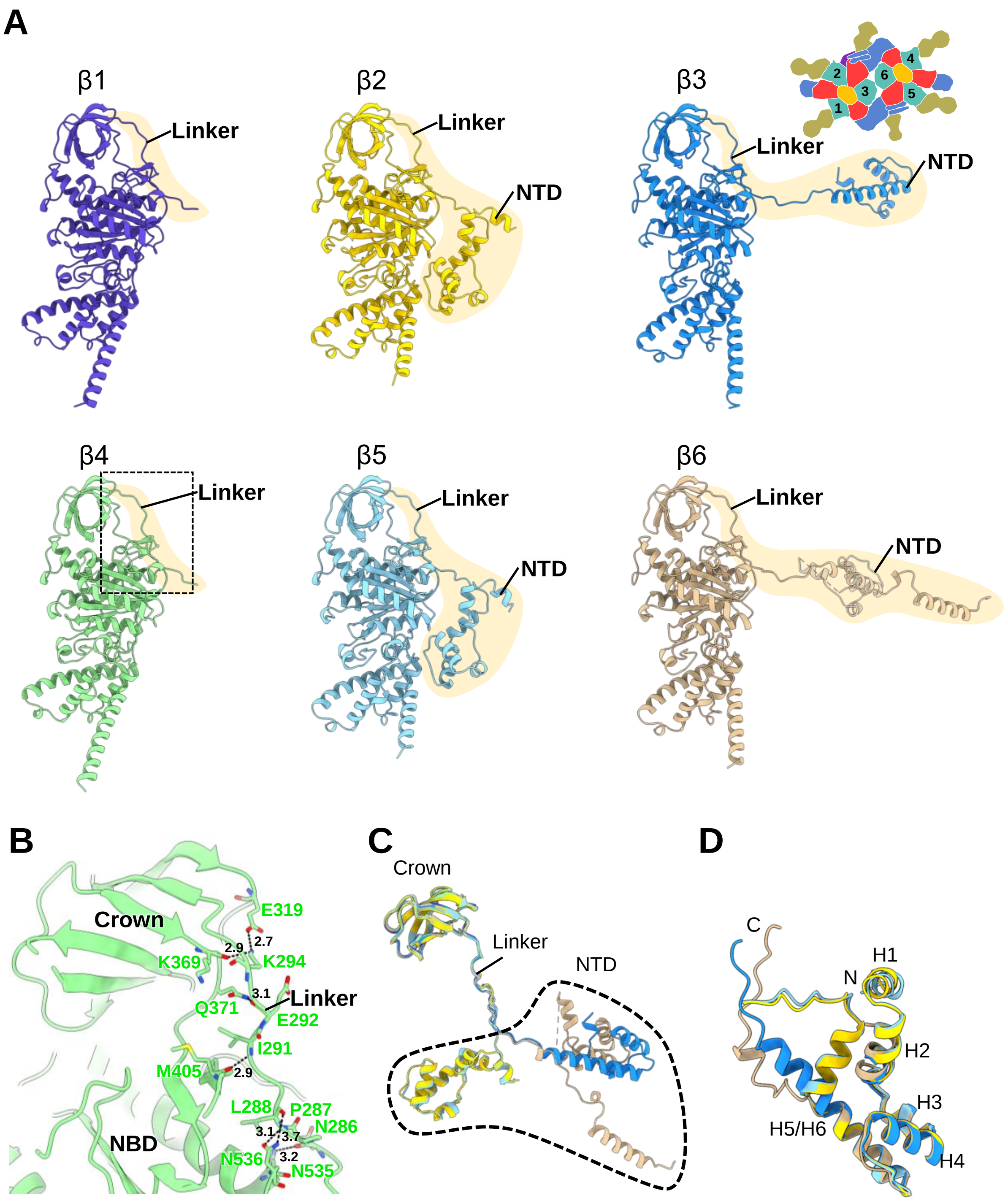

Fig. S9. Extended N-terminal region of β.

(**A**) Structures of β1–6 in twin motor. Extended N-terminal regions are marked in khaki. An illustration with the location of each β is presented in the upper right corner. (**B**) Linker interactions with crown and NBD in β4.The region corresponds to the black dot box in (A). (**C**) Superposition of structures composed of crown, linker, and NTD. Structures were superimposed on the crown. (**D**) Superposition of NTD.

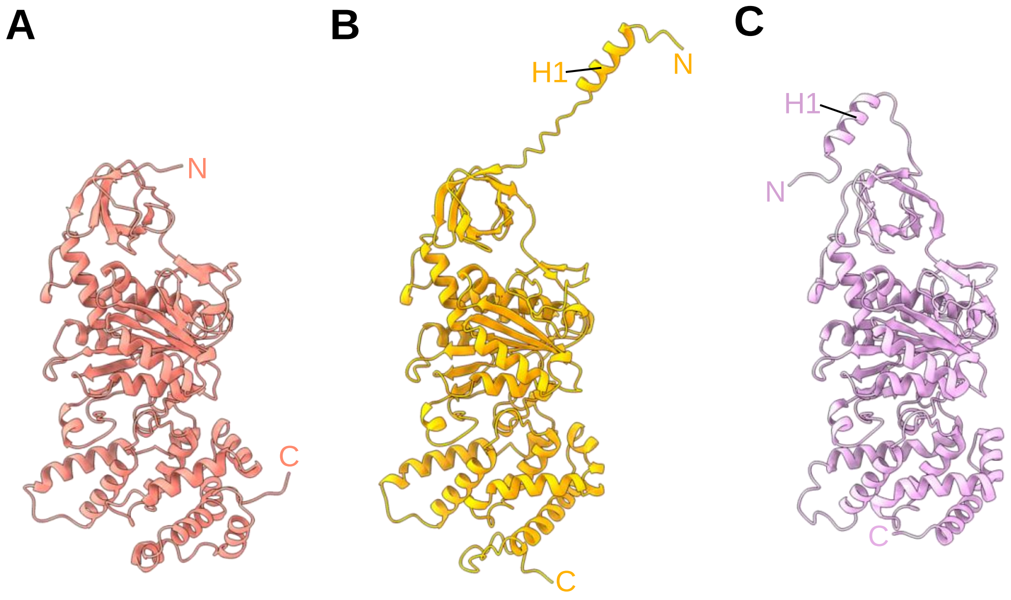

Fig. S10. N-terminal helix (H1) of F_1-ATPase α not found in G_1-α.
(**A**) G_1-α2 structure. (**B**) Structure of F_1-ATPase (Type 1 ATPase) α from *M. mobile* predicted using AlphaFold2. (**C**) Structure of F_1-ATPase α from *Bacillus* PS3 (PDB ID: 6N2Y).

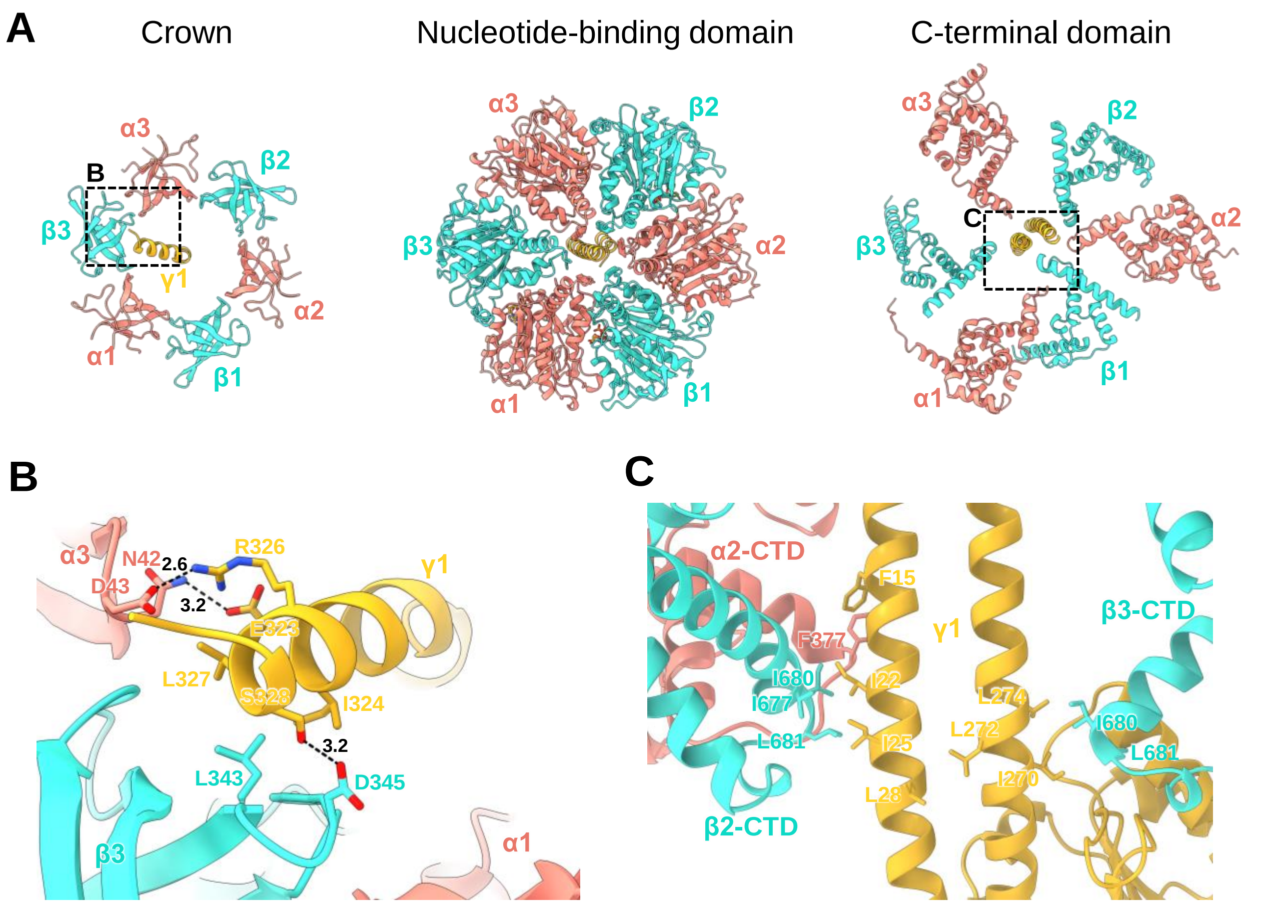

Fig. S11. Asymmetry of G_1-ATPase and interaction between hexameric ring and γ.
(**A**) Cross-section of each domain of $\alpha_3\beta_3\gamma$ subcomplex. (**B**) Interaction between the crown region of the hexameric ring and extended helix of γ. The region corresponds to the black dot box in (A). (**C**) Hydrophobic interaction between the C-terminal domain of the hexameric ring and coiled-coil of γ. The region corresponds to the black dot box in (A).

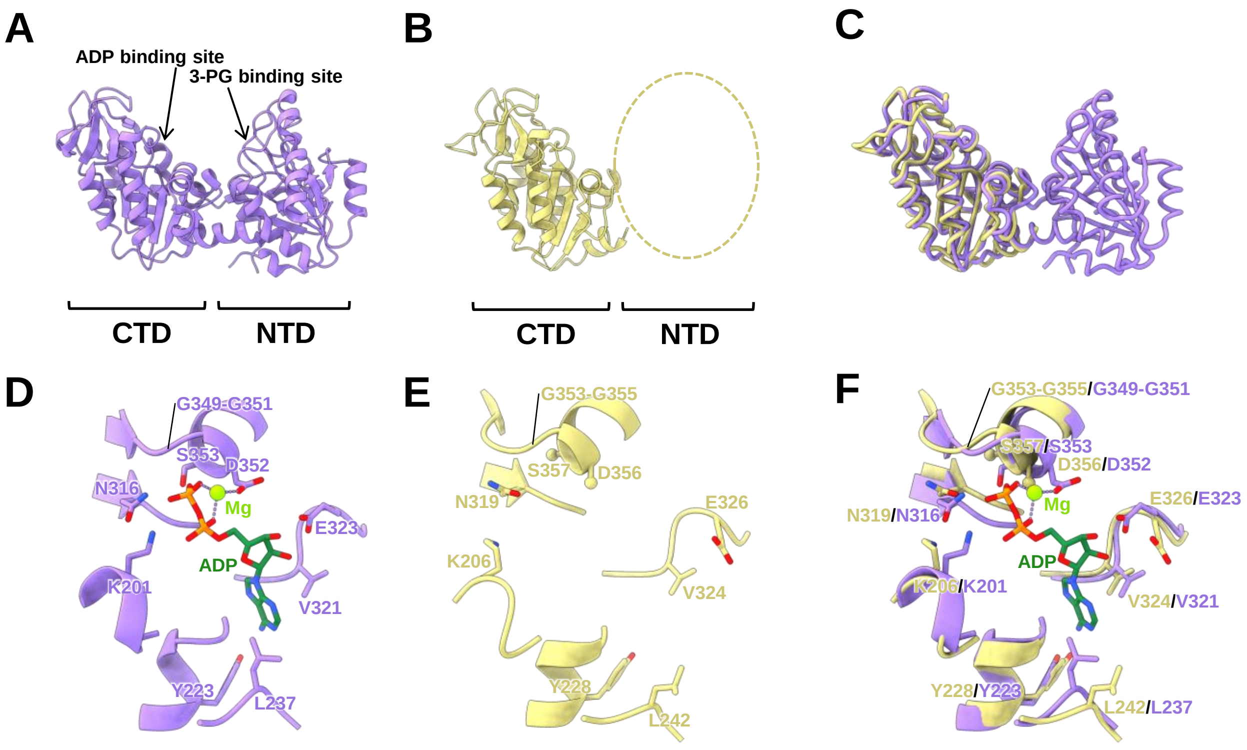

Fig. S12. Comparison of the PGK structure.
(**A**) PGK structure from *S. aureus* (PDB ID: 4DG5). (**B**) PGK structure in the twin motor. (**C**) Superimposition of the PGK molecules. (**D**) Catalytic site binding Mg-ADP in PGK (PDB ID: 1PHP). (**E**) Catalytic site of PGK in the twin motor. (**F**) Superimposition of the catalytic sites.

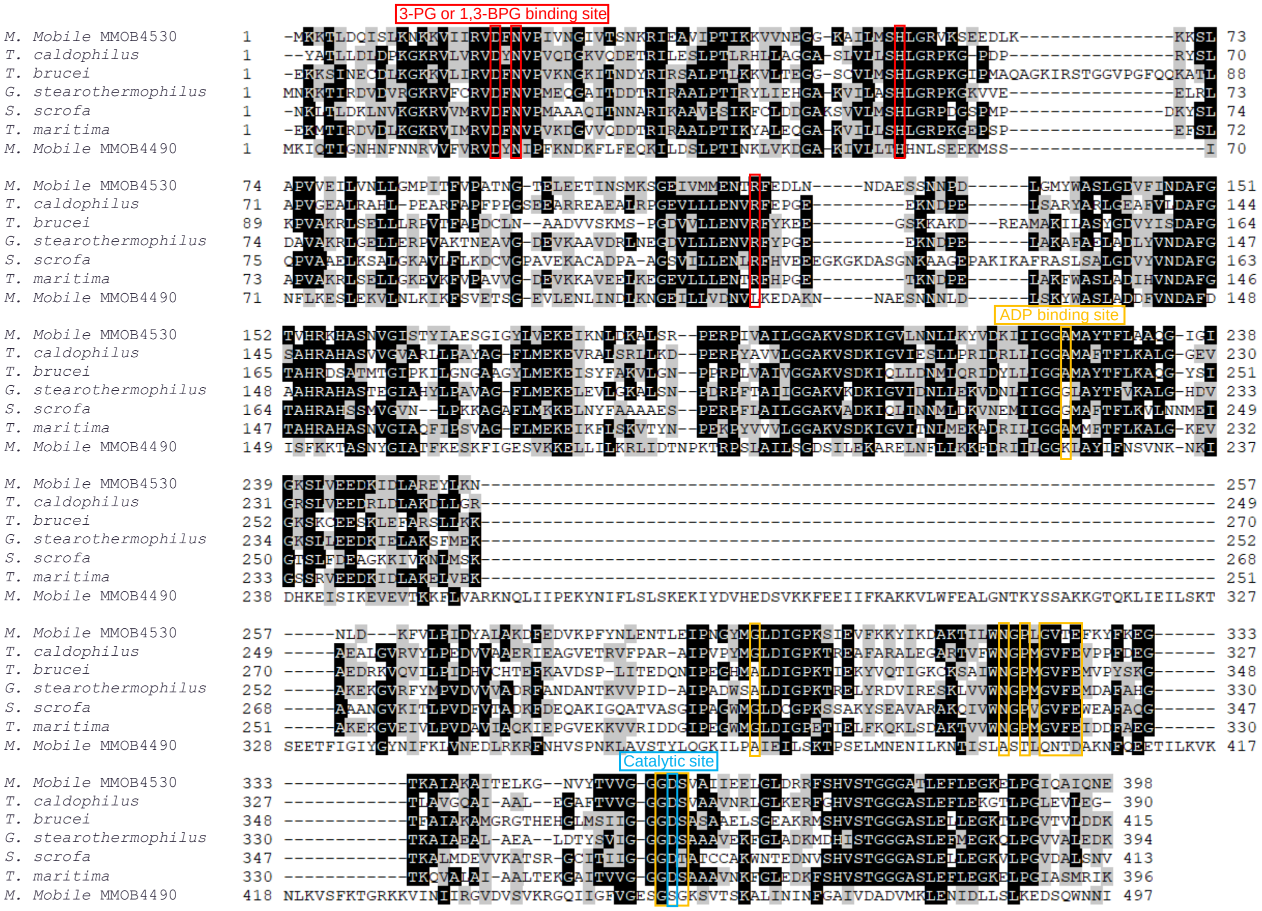

Fig. S13. Amino acid sequence alignment of PGK and the homologue.
ADP binding, catalytic, and 3-PG or 1,3-BPG binding sites are indicated by orange, blue, and red boxes, respectively. Amino acid sequences of PGKs and the homologue were aligned using ClustalW. The figure was generated using BioEdit.

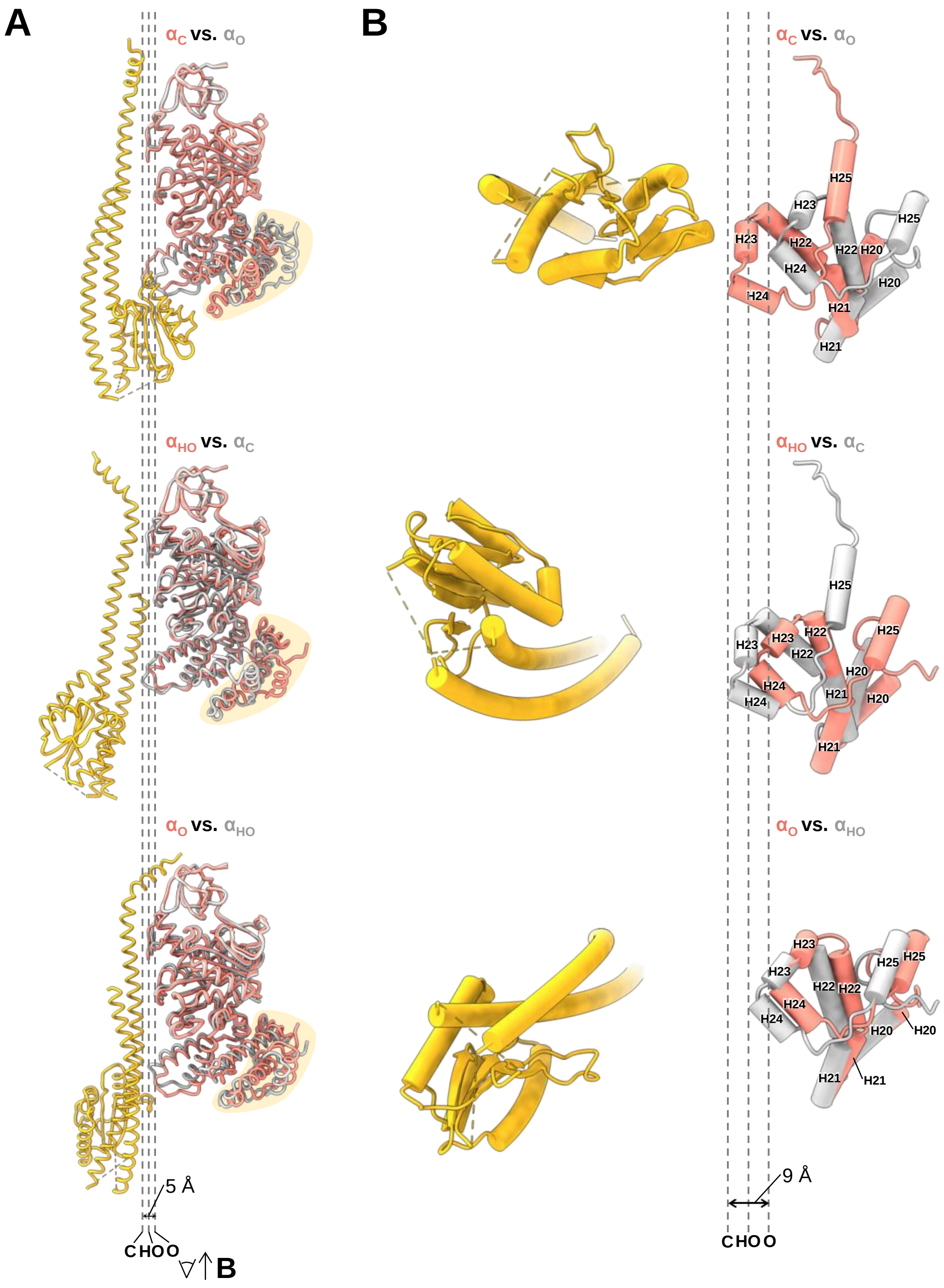

Fig. S14. Differences in the conformations of the three α.
(**A**) Superposition of three α in the G_1-ATPase. The dotted lines indicate the position of the region corresponding to DELSEED loop in each α. H20–H25 regions are marked in khaki. (**B**) Comparison of the H20–H25 region among the three α. Each image is a bottom-view of (A).

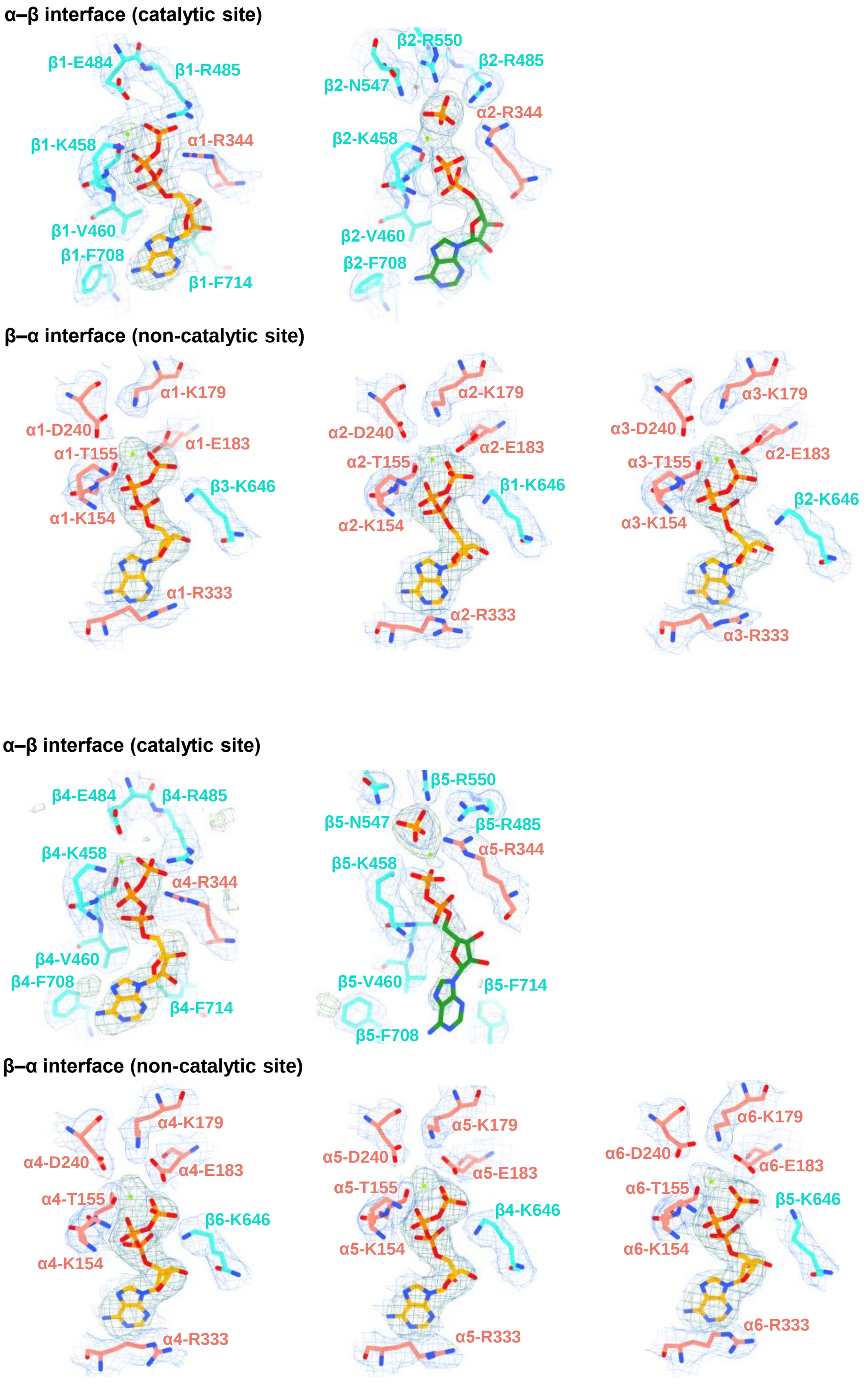

Fig. S15. Maps of the nucleotide binding sites of G_1-ATPases.

F_o maps and F_o-F_c maps are displayed by blue and green nets. The F_o-F_c maps are contoured at 5.50 except for the α4β4 (3.30) and α5β5 (4.00) interfaces, while the Fo maps are contoured at 0.55 in UCSF ChimeraX.

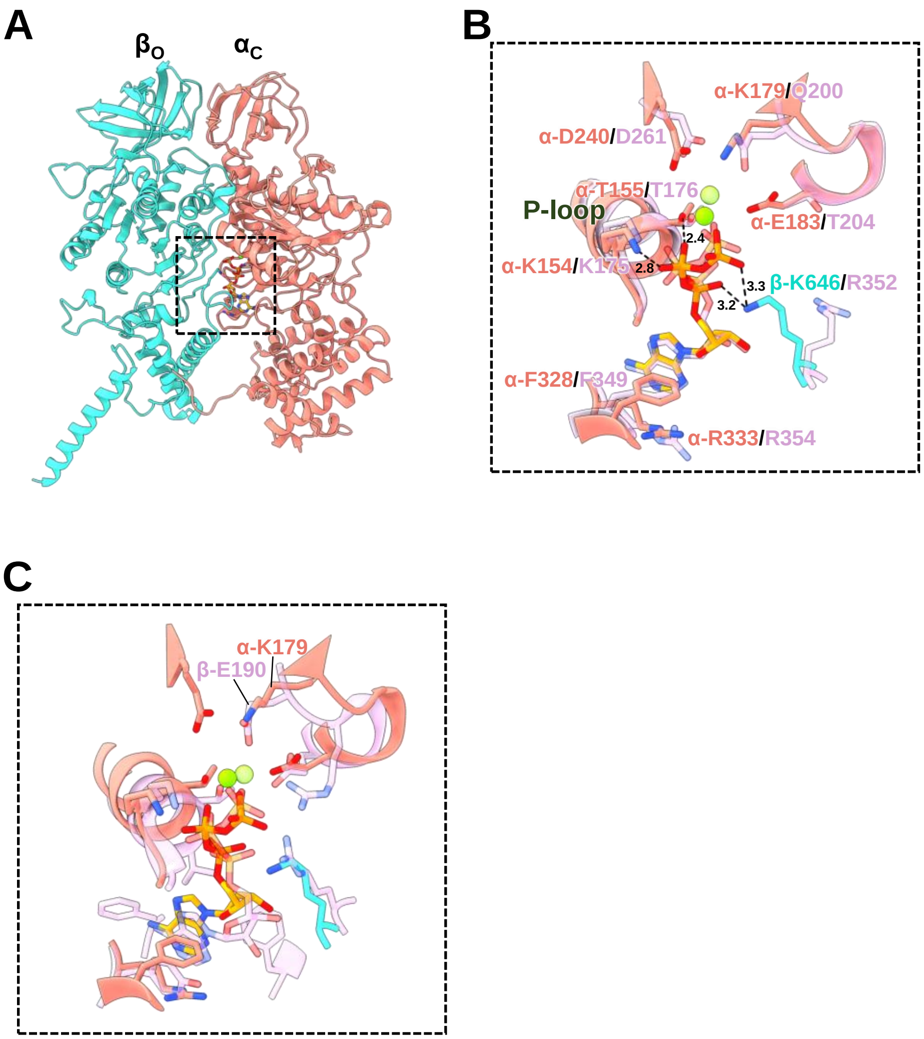

Fig. S16. Interface corresponding to the non-catalytic site of F_1-ATPase.
(**A**) Interface formed by G_1-β_O and G_1-α_C. (**B**, **C**) Comparison of the interface between the F_1-ATPase and G_1-ATPase. The interface from the dot square in (A) is superimposed on the corresponding non-catalytic site (B) and catalytic site (C) of F_1-ATPase (PDB ID: 8HHA), coloured purple.

	Twin motor (EMD-60718) (PDB ID: 9IO5)
**Data collection and processing**	
Microscope	CRYO ARM 300
Detector	K3
Nominal magnification	60,000×
Voltage (kV)	300
Total electron exposure (e^-/Å^2)	80
Frames (no.)	40
Total exposure time (sec)	3.3
Defocus range (µm)	−0.8 to −1.8
Pixel size (Å)	0.87
Total image sets (no.)	7,350
Used image sets (no.)	7,074
Initial particle images (no.)	633,820
Final particle images (no.)	142,490
Symmetry imposed	C1
Map resolution (Å)	3.2
B-factor for sharpening (Å^2)	−57.3
FSC threshold	0.143
**Model refinement**	
Initial models used	Homology models and AlphaFold2-predicted structures
Model resolution	3.2
FSC threshold	0.5
Model composition	
Non-hydrogen atoms (no.)	69,678
Protein residues (no.)	8,930
Ligands (no.)	22
B factors	
Protein (Å^2)	35.37
Ligands (Å^2)	34.50
R.m.s.deviations	
Bond lengths (Å^2)	0.004
Bond angles (˚)	0.944
Valiation	
Molprobity score	1.48
Clashscore	5.46
Poor rotamers (%)	0.00
Ramachandran plot	
Favored (%)	96.91
Allowed (%)	3.06
Disallowed (%)	0.03

Table S1. Cryo-EM data collection and model statistics.

Chain ID	Protein	Protein length (amino acids)	Modelled amino acid residues	Q-score
A	β1	784	281–778	0.60
B	β2	784	202–771	0.59
C	β3	784	217–777	0.57
D	α1	528	1–528	0.61
E	α2	528	1–528	0.58
F	α3	528	1–528	0.60
G	γ1	336	1–56, 76–190, 243–331	0.49
H	β4	784	281–779	0.60
I	β5	784	201–771	0.57
J	β6	784	125–159, 213–774	0.58
K	α4	528	1–528	0.60
L	α5	528	1–528	0.58
M	α6	528	1–528	0.60
N	γ2	336	1–57, 77–190, 247–331	0.48
O	PGK1	511	179–344, 348–385	0.37
P	PGK2	511	179–365, 370–385	0.34
Q	PGK3	511	179–343, 349–386	0.33
R	GliD1	293	47–53, 58–270	0.47
S	GliD2	293	109–194, 207–249, 255–272, 277–287	0.36
T	GliD3	293	62–290	0.55
U	GliD4	293	45–270	0.50
V	GliD5	293	106–195, 207–288	0.38
W	GliD6	293	65–290	0.52
X	GliE	112	4–112	0.59
Y	Unknown helix		1–13	0.45
Z	Unknown helix		1–9	0.35

Table S2. Calculated Q-score for each subunit.

	0˚ (*ATP-waiting*)	0˚ (*step-waiting*)	81˚	83˚ (*post-hyd*)	91˚	101˚
β1 (C)	2.05	2.16	2.18	2.15	2.15	2.16
β2 (HO)	1.95	2.26	3.36	2.67	2.67	2.03
β3 (O)	2.19	2.18	2.33	2.33	2.33	2.35

Table S3. RMSD values (Å) between G_1-β and *Bacillus* PS3 F_1-ATPase β.
G_1-β1–3 (C, HO, and O) were superimposed on the same conformation of the β subunit in the six states of *Bacillus* PS3 F_1-ATPase (PDB ID: 8HH1–8HH6), respectively. The state names of *Bacillus* PS3 F_1-ATPase are based on the γ subunit position (*16*). The lowest RMSD value for each G_1-β is shown in red.
